# Effects of strengthening the surae triceps muscle on venous pump function in chronic venous insufficiency

**DOI:** 10.1590/1677-5449.200197

**Published:** 2021-08-16

**Authors:** Ana Carla Schmidt, Luisa Pereira de Oliveira Zanetti Gomes, Camila Martins Marinelli, Ricardo Zanetti Gomes

**Affiliations:** 1 Centro de Ensino Superior dos Campos Gerais, Ponta Grossa PR, Brasil.; 2 Universidade Estadual de Ponta Grossa – UEPG, Ponta Grossa PR, Brasil.; 3 Universidade da Região de Joinville – UNIVILLE, Joinville, SC, Brasil.

**Keywords:** chronic venous insufficiency, physical exercise, physical activity

## Abstract

**Background:**

Chronic venous insufficiency (CVI) is a common disease that causes calf muscle pump dysfunction and has repercussions for the hemodynamics of the structures involved.

**Objectives:**

To analyze the effects on venous hemodynamics of exercises to strengthen the calf muscles in patients with CVI.

**Methods:**

The study analyzed 25 lower limbs with CVI, classified from C1 to C5 according to the Clinical, Etiology, Anatomy, and Pathophysiology (CEAP) classification, in 13 patients recruited from a Lymphedema and Angiodysplasia Clinic at the Hospital Universitário Regional dos Campos Gerais (Brazil). The variables analyzed were collected by isometric dynamometry, goniometry, leg circumference measurement, and adipometry at baseline, after 1 month and 2 months and at the end of the exercise protocol.

**Results:**

Dorsiflexion and plantar flexion measurements increased by 5º (p < 0.001). Adipometry detected a reduction in 5 mm (p < 0.001). When grouped by CEAP class, C2 exhibited 5º increases in dorsiflexion (p = 0.02) and plantar flexion (p < 0.001); C3 exhibited a 5ºincrease in dorsiflexion (p = 0.003) and a 1mm reduction in adipometry (p < 0.004); and C1 exhibited a 1.2cm increase in leg circumference (p = 0.04).

**Conclusions:**

Administration of exercise protocols should be considered as a treatment option for CVI, since it has a positive impact on risk factors and on the functions that are impaired by this pathology.

## INTRODUCTION

Chronic venous insufficiency (CVI) is a progressive disease characterized by abnormalities affecting the flow of blood through the veins. It develops as a result of a combination of venous reflux, venous obstruction, and an ineffective calf muscle pump. The veins in the calf and the tissues that surround them form the muscle pump, which is involved in drainage of venous blood and is activated by movement of the ankle joint. Calf muscle pump dysfunction can aggravate CVI, causing venous hypertension.^1^^,^^2^

There are currently a wide range of CVI treatments intended to prevent sequelae and improve symptoms and complications. Treatment approaches include surgical procedures, compression therapy, and pharmacological treatments. To complement these options, physical exercise has been studied as a method for prevention and rehabilitation of CVI, alleviating the burden on public health systems by reducing expenditure and increasing these patients’ productivity.^3^^-^5

The effects of structured exercise programs for patients with venous dysfunction include improved muscle function, increasing strength, trophic variables, and venous hemodynamics. Such exercise programs generally consist of techniques for stretching and strengthening the muscles of the lower limbs, combined with aerobic exercises.^6^^-^8 The objective of this study was therefore to analyze the effects on venous hemodynamics of exercises to strengthen the muscles of the calf, comparing the improvements at different points in time over the course of the intervention and between different groups staged according to the Clinical, Etiology, Anatomy, and Pathophysiology (CEAP) classification.

## METHODS

This is an experimental study with a non-randomized clinical trial design and no control group, investigating a population with CVI. It was conducted at a Lymphedema and Angiodysplasia Clinic where patients with CVI receive medical care at the Hospital Universitário Regional dos Campos Gerais and was approved by the institutional Ethics Committee (ruling number: 3653067). Patients of both sexes with CVI classified as CEAP grades C1 to C5 who had not undergone conventional varicose vein surgery or foam sclerotherapy were included if they had achieved at least 60% attendance in the calf strengthening group sessions. Individuals were excluded if they had lower limb disorders of orthopedic, rheumatic, and/or neurological etiologies or venous ulcers, a recent history of lower limb fractures, or if they did not consent to participation.

### Recruitment of the sample

**Figure 1:**
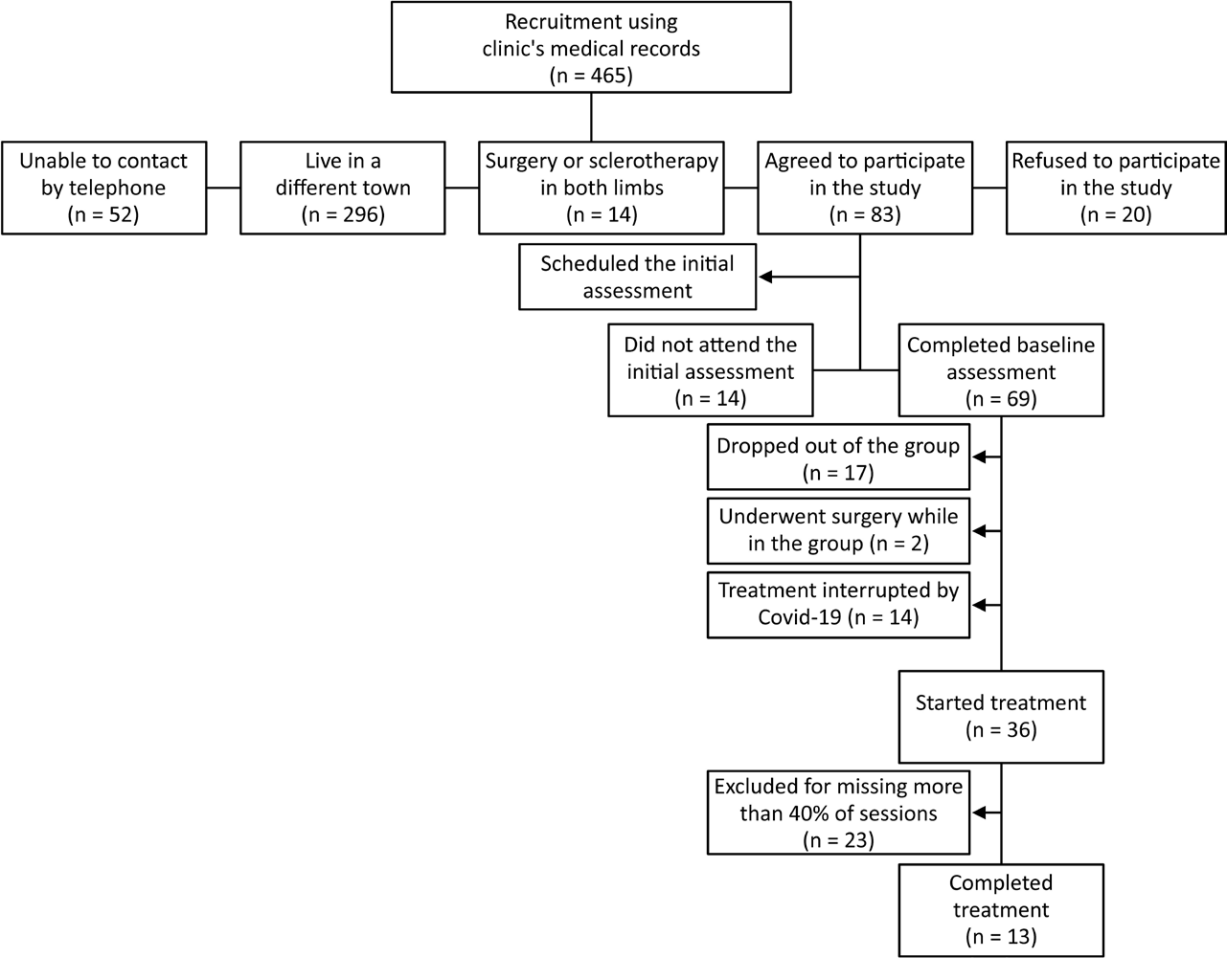
Sample recruitment.

A sample size calculation was performed considering an estimated effect of 20%, 80% power, and 10% error, resulting in a minimum sample of 42 limbs. The vascular clinic’s medical records were then searched and pre-selected patients were contacted by telephone (Figure 1). A form was drawn up by the study authors to collect data on the principal characteristics of the study population and information about their disease. This form was administered to the 69 participants initially identified as eligible and the physical examination was repeated for the 13 patients who completed all phases of the study, totaling 25 lower limbs analyzed (one limb was excluded from the analysis after undergoing surgery). At this point, a sample power calculation for the same 20% predicted rate of success and a sample of 25 limbs resulted in a sample power of 64%.

### Physical examinations

The patients selected underwent tests of triceps surae muscle strength using a Manual Muscle Test 01165® portable isometric dynamometer. For these measurements, the patient was placed in the supine position, with hips and knees extended and ankles in a neutral position, with the dynamometer positioned against the sole of the foot at the proximal portion of the metatarsal joints. In this position, the patient was instructed to perform plantar flexion against the examiner’s resistance. Measurements were repeated three times at 15-second intervals and the mean was calculated, with the result expressed in kilograms-force.

Ankle joint amplitude was measured by manual goniometry, taking the result of the sum of the maximum voluntary plantar flexion and dorsiflexion from the neutral mid-point at 90º. The expected values are 45º for plantar flexion and 20º for dorsiflexion, making a total amplitude of 65º. Both dorsiflexion and plantar flexion measurements were taken with the patient lying supine with the knees flexed at 30º and the foot in the anatomical position.

Leg circumference was measured with a tape measure with the patient standing upright, marking points at 10 cm intervals from the lower margin of the patella to the malleolus for both lower limbs. Measurements were taken during the afternoon and the unit of measurement employed was centimeters.

Adipometry was employed to determine the fat percentage at the calf. The evaluator pinched a skin fold with the fingers around 1.5 cm from the point where the adipometer was applied, which was at the point of maximum leg circumference with the patient seated. The measurement was repeated three times and the mean calculated in millimeters.

Data were collected individually at the start of the intervention (1st session), 1 month after starting the intervention (9th session), 2 months after starting the intervention (17th session), and at the end of the intervention (25th session).

### Exercise protocol

The intervention duration was 3 months, over a total of 24 sessions: two sessions per week, of 30 minutes each, performing three to five series of 10 to 12 repetitions of each exercise, with 60 second intervals between series, according to training parameters described by Schoenfeld.^9^

Both open and closed kinetic chain exercises were performed, involving plantar and dorsal flexion of the ankle joint. Each movement followed the sequence of concentric, eccentric, and then isometric movements, which intensify the exercise.

### Statistical analysis

Initially, a descriptive analysis of patient variables was conducted, estimating simple frequencies. The dynamometry, dorsiflexion, plantar flexion, leg circumference, and adipometry results were analyzed with estimation of mean, median, standard deviation, and 25th and 75th percentiles for each data collection period.

Next, the Shapiro-Wilk test was used to determine whether variables conformed to the normal distribution and, since they did not, nonparametric data analysis methods were used. Differences between different data collection times were tested with the Friedman test followed by multiple post-hoc comparisons with Bonferroni correction. Tests were considered significant if p < 0.05 and analyses were performed using SPSS 21.0.^10^

## RESULTS

In this study, 13 participants (25 lower limbs) completed 24 sessions of the physiotherapy intervention and attended four assessment consultations. The sample comprised women aged 38 to 70 years who had been diagnosed with CVI for a mean of 6.4 (standard deviation: 7.5) years. Table 1 lists data used to identify the participants, associated pathologies, medications used, and lifestyle details.

**Table 1:** Sample characteristics.

**Characteristics**			**n**	**%**
Sex	Female		13	100.0%
Age group	< 50 years		6	46.2%
	> 50 years		7	53.8%
Times since diagnosis	< 5 years		9	69.2%
	< 5 years		4	30.8%
Compression stockings	Yes		6	46.2%
	No		7	53.8%
Comorbid pathologies	Diabetes mellitus		5	38.5%
	Arterial hypertension		4	30.8%
	Thyroid disorders		3	23.1%
	Dyslipidemia		2	15.4%
	Others		7	53.8%
Medications used	Diosmin + hesperidin		5	38.5%
	Levothyroxine		5	38.5%
	Metformin		3	23.1%
	Losartan		2	15.4%
	Simvastatin		2	15.4%
	Acetylsalicylic acid		2	15.4%
	Omeprazole		2	15.4%
	Others		15	115.5%
Lifestyle	Smoking	Yes	1	7.7%
		No	8	61.5%
		Ex-smoker	4	30.8%
	Alcoholism	No	12	92.3%
		Ex-alcoholic	1	7.7%
	Physical activity	Sedentary	7	53.8%
		Active	6	46.2%
	Frequency of physical activity	1 day	1	7.7%
		3 days	3	23.1%
		7 days	2	15.4%

Each patient’s venous disease was graded according to the CEAP classification. Participants were grouped by their clinical (C) grade, as shown in Table 2, which lists the number of lower limbs in each grade. Analyzing these groups formed on the basis of the CEAP classification, it was observed that there were no statistically significant intergroup or intragroup differences in increase in triceps surae muscle strength, as shown in Table 3. However, analysis of the overall dynamometry results (Table 4) showed that strength had increased from the first to the last assessment, although the increase was not significant.

**Table 2:** CEAP classification of CVI.

CEAP	n	%
**C1**	5	20.0%
**C2**	11	44.0%
**C3**	7	28.0%
**C4**	1	4.0%
**C5**	1	4.0%

CEAP = Clinical, Etiology, Anatomy, and Pathophysiology.

**Table 3:** Medians and interquartile ranges for dynamometry results from patients who performed supervised exercise for 3 months, with p values for tests for differences.

**CEAP**	**n**	**Dynamometry - kgf**												***p*-value between times** *
		**Baseline**			**1 month**			**2 months**			**Final**			
		**MD**	**Perc. 25%**	**Perc. 75%**	**MD**	**Perc. 25%**	**Perc. 75%**	**MD**	**Perc. 25%**	**Perc. 75%**	**MD**	**Perc. 25%**	**Perc. 75%**	
C1	5	7.3	6.9	8.0	7.5	7.5	8.2	7.8	7.7	7.8	7.5	7.3	7.8	0.246
C2	11	7.3	6.9	9.8	8.2	7.7	8.9	8.0	7.6	8.8	8.7	7.6	8.8	0.383
C3	7	9.3	6.8	10.1	8.2	6.3	9.2	8.7	7.0	10.0	9.0	7.5	9.6	0.136
C4	1	8.4	8.4	8.4	10.4	10.4	10.4	7.3	7.3	7.3	8.1	8.1	8.1	0.392
C5	1	8.4	8.4	8.4	7.6	7.6	7.6	10.0	10.0	10.0	8.5	8.5	8.5	0.392

MD = median; Perc. = percentile.

* Friedman test (analogous to a nonparametric ANOVA for repeated measures). Different letters indicate statistically significant differences in two by two comparisons.

**Table 4:** Overall medians and interquartile ranges for patients who performed supervised exercise for 3 months, with p values for tests for differences.

**Overall**	**n**	**Baseline**			**1 month**			**2 months**			**Final**			***p-*value between times** *
		**MD**	**Perc. 25%**	**Perc. 75%**	**MD**	**Perc. 25%**	**Perc. 75%**	**MD**	**Perc. 25%**	**Perc. 75%**	**MD**	**Perc. 25%**	**Perc. 75%**	
Dynamometry (kgf)	25	8.0a	6.9	9.8	8.2a	7.5	8.9	8.0a	7.6	8.9	8.5a	7.5	8.8	0.266
Dorsiflexion (º)	25	15.0a	10.0	20.0	20.0ab	20.0	20.0	20.0b	20.0	20.0	20.0b	20.0	25.0	< 0.001
Plantar flexion (º)	25	40.0a	35.0	40.0	40.0a	40.0	40.0	40.0a	40.0	45.0	45.0b	42.0	50.0	< 0.001
Leg circumference (cm)	25	30.2ª	29.3	33.0	31.8a	29.3	33.3	31.8a	29.0	33.3	30.8a	29.2	33.2	0.215
Adipometry (mm)	25	35.0a	30.0	45.0	32.0ab	30.0	40.0	30.0ab	30.0	40.0	30.0b	30.0	40.0	< 0.001

MD = median; Perc. = percentile.

* Friedman test (analogous to a nonparametric ANOVA for repeated measures). Different letters indicate statistically significant differences in two by two comparisons.

The same analysis was conducted with goniometry results separated into dorsiflexion and plantar flexion. The dorsiflexion results showed that groups C2 (n = 11) and C3 (n = 7) benefited more from the exercises than the other groups (Table 5). These results were also observed for plantar flexion, as shown in Table 6. When these variables were analyzed without dividing the sample into subsets (Table 5), it was observed that movement amplitude increased significantly from the first to the last session (p < 0.001, both).

**Table 5:** Medians and interquartile ranges for dorsiflexion goniometry results from patients who performed supervised exercise for 3 months, with p values for tests for differences.

**CEAP**	**n**	**Dorsiflexion (º)**												***p*-value between times** *
		**Baseline**			**1 month**			**2 months**			**Final**			
		**MD**	**Perc. 25%**	**Perc. 75%**	**MD**	**Perc. 25%**	**Perc. 75%**	**MD**	**Perc. 25%**	**Perc. 75%**	**MD**	**Perc. 25%**	**Perc. 75%**	
C1	5	20.0	20.0	20.0	25.0	20.0	25.0	25.0	20.0	25.0	25.0	20.0	30.0	0.072
C2	11	15.0	10.0	20.0	20.0	20.0	20.0	20.0	20.0	20.0	20.0	20.0	25.0	0.002
C3	7	15.0	10.0	20.0	20.0	20.0	20.0	20.0	20.0	20.0	20.0	20.0	25.0	0.002
C4	1	10.0	10.0	10.0	15.0	15.0	15.0	15.0	15.0	15.0	20.0	20.0	20.0	0.392
C5	1	10.0	10.0	10.0	10.0	10.0	10.0	10.0	10.0	10.0	13.0	13.0	13.0	0.392

MD = median; Perc. = percentile.

* Friedman test (analogous to a nonparametric ANOVA for repeated measures). Different letters indicate statistically significant differences in two by two comparisons.

**Table 6:** Medians and interquartile ranges for plantar flexion goniometry results from patients who performed supervised exercise for 3 months, with p values for tests for differences.

**CEAP**	**n**	**Plantar flexion (º)**												***p-*value between times** *
		**Baseline**			**1 month**			**2 months**			**Final**			
		**MD**	**Perc. 25%**	**Perc. 75%**	**MD**	**Perc. 25%**	**Perc. 75%**	**MD**	**Perc. 25%**	**Perc. 75%**	**MD**	**Perc. 25%**	**Perc. 75%**	
C1	5	40.0	30.0	40.0	40.0	35.0	40.0	40.0	35.0	40.0	40.0	40.0	40.0	0.067
C2	11	40.0	35.0	40.0	40.0	40.0	45.0	40.0	40.0	45.0	45.0	45.0	50.0	< 0.001
C3	7	40.0	30.0	40.0	40.0	40.0	40.0	40.0	40.0	40.0	45.0	45.0	50.0	0.003
C4	1	45.0	45.0	45.0	45.0	45.0	45.0	45.0	45.0	45.0	50.0	50.0	50.0	0.392
C5	1	35.0	35.0	35.0	40.0	40.0	40.0	45.0	45.0	45.0	50.0	50.0	50.0	0.392

MD = median; Perc. = percentile.

* Friedman test (analogous to a nonparametric ANOVA for repeated measures). Different letters indicate statistically significant differences in two by two comparisons.

In contrast with the results reported so far, it was observed (Table 7) that the leg circumference measurements had increased significantly in the C1 group (n = 5) at the end of the 24 sessions compared with the other groups. Analysis of the overall results for leg circumference (Table 4), without subdivision into CEAP groups, revealed that measurements had increased, but that the difference was not statistically significant.

**Table 7:** Medians and interquartile ranges for leg circumference measurements from patients who performed supervised exercise for 3 months, with p values for tests for differences.

	**n**	**Leg circumference (cm)**												***p*-value between times** *
		**Baseline**			**1 month**			**2 months**			**Final**			
		**MD**	**Perc. 25%**	**Perc. 75%**	**MD**	**Perc. 25%**	**Perc. 75%**	**MD**	**Perc. 25%**	**Perc. 75%**	**MD**	**Perc. 25%**	**Perc 75%**	
C1	5	29.5	29.1	32.2	31.8	28.8	32.7	31.8	29.3	32.7	30.7	28.8	31.7	0.045
C2	11	29.8	29.0	31.5	30.3	29.3	32.7	29.2	28.5	33.0	29.3	29.2	32.2	0.197
C3	7	32.7	30.2	33.7	33.3	30.0	33.8	33.3	29.8	33.5	32.0	30.3	33.5	0.427
C4	1	29.3	29.3	29.3	29.2	29.2	29.2	29.2	29.2	29.2	28.5	28.5	28.5	0.392
C5	1	38.7	38.7	38.7	38.7	38.7	38.7	38.8	38.8	38.8	38.8	38.8	38.8	0.392

MD = median; Perc. = percentile.

* Friedman test (analogous to a nonparametric ANOVA for repeated measures). Different letters indicate statistically significant differences in two by two comparisons.

The adipometry results for group C3 showed a significant reduction (p = 0.004). The measurements for group C4 also reduced, but the difference was not significant (Table 8). It was observed that the percentage calf fat of all 25 limbs had reduced at the end of the intervention (Table 4) (p < 0.001).

**Table 8:** Medians and interquartile ranges for adipometry results from patients who performed supervised exercise for 3 months, with p values for tests for differences.

**CEAP**	**n**	**Adipometry (mm)**												***p*-value between times** *
		**Baseline**			**1 month**			**2 months**			**Final**			
		**MD**	**Perc. 25%**	**Perc. 75%**	**MD**	**Perc. 25%**	**Perc. 75%**	**MD**	**Perc. 25%**	**Perc. 75%**	**MD**	**Perc. 25%**	**Perc. 75%**	
C1	5	35.0a	30.0	40.0	32.0a	30.0	40.0	32.0a	30.0	40.0	40.0a	30.0	40.0	0.194
C2	11	30.0a	26.0	35.0	30.0a	25.0	35.0	30.0a	25.0	32.0	30.0a	25.0	32.0	0.153
C3	7	36.0a	35.0	51.0	36.0ab	35.0	45.0	35.0ab	30.0	48.0	35.0b	30.0	40.0	0.004
C4	1	25.0a	25.0	25.0	20.0a	20.0	20.0	20.0a	20.0	20.0	18.0a	18.0	18.0	0.392
C5	1	50.0a	50.0	50.0	50.0a	50.0	50.0	40.0a	40.0	40.0	50.0a	50.0	50.0	0.392

MD = median; Perc. = percentile.

* Friedman test (analogous to a nonparametric ANOVA for repeated measures). Different letters indicate statistically significant differences in two by two comparisons.

Analysis of the statistically significant results reveals differences between different assessment times in the dorsiflexion, plantar flexion, and adipometry variables. Dorsiflexion was significantly greater at 2 months and at the end of the intervention (p = 0.016 and p < 0.001, respectively). Plantar flexion was significantly greater at the last assessment than at baseline, 1 month, and 2 months (p < 0.001, p = 0.003 and p = 0.037, respectively). Adipometry results were significantly lower at the last assessment than at baseline (p = 0.03). The confidence intervals for the study findings are shown in the Table 9.

**Table 9:** Table of confidence intervals for variables analyzed.

	**Baseline**			**1 month**			**2 months**			**Final**		
	**M**	**95%CI**		**M**	**95%CI**		**M**	**95%CI**		**M**	**95%CI**	
		**L**	**U**		**L**	**U**		**L**	**U**		**L**	**U**
**OVERALL**												
Dynamometry	8.4	7.7	9	8.3	7.7	8.8	8.4	7.9	9.0	8.4	8	8.8
Dorsiflexion	15.8	13.4	18.2	19.6	17.8	21.4	20.4	18.8	22	22.4	20	24.8
Plantar flexion	37.6	35.7	39.5	40.4	38.6	42.2	41.4	39.8	43	45.3	43.5	47.1
Leg circumference	31.6	30.3	32.9	31.9	30.7	33.1	31.6	30.3	32.9	31.5	30.2	32.7
Adipometry	35.8	31.6	39.9	34.1	30.8	37.5	33.4	30.1	36.6	33.7	30.2	37.3
**CEAP 1**												
Dynamometry	8.0	5.9	10.0	8.4	6.1	10.6	8.5	6.1	10.9	8.0	6.4	9.6
Dorsiflexion	21.0	14.2	27.8	24.0	18.8	29.2	24.0	18.8	29.2	27.0	16.6	37.4
Plantar flexion	36.0	29.2	42.8	37.0	31.4	42.6	38.0	30.9	45.1	40.0	35.6	44.4
Leg circumference	30.7	27.2	34.3	31.3	27.7	34.9	31.4	27.9	34.9	30.6	27.4	33.8
Adipometry	36.0	27.9	44.1	34.4	28.0	40.8	34.4	28.0	40.8	36.6	29.0	44.2
**CEAP 2**												
Dynamometry	8.2	7.0	9.5	8.3	7.8	8.7	8.2	7.6	8.8	8.4	7.9	8.9
Dorsiflexion	15.5	11.6	19.3	19.1	16.6	21.6	20.5	18.6	22.3	21.8	18.4	25.3
Plantar flexion	38.2	35.9	40.4	41.8	38.4	45.3	42.7	40.4	45.0	45.9	43.4	48.4
Leg circumference	31.1	28.9	33.3	31.4	29.4	33.4	30.9	28.8	33.0	31.1	29.0	33.2
Adipometry	31.1	25.8	36.4	30.5	26.4	34.6	30.2	26.2	34.2	30.8	25.1	36.6
**CEAP 3**												
Dynamometry	8.8	7.4	10.3	7.9	6.5	9.3	8.7	7.2	10.2	8.7	7.7	9.7
Dorsiflexion	14.3	10.1	18.4	19.2	17.5	21.0	20.0	*	*	21.7	19.5	23.9
Plantar flexion	37.1	31.9	42.4	40.0	*	*	40.7	39.0	42.5	46.7	43.7	49.7
Leg circumference	32.3	29.9	34.5	32.6	30.4	34.7	32.2	29.9	34.4	32.0	29.8	34.2
Adipometry	42.4	32.1	52.7	39.4	32.7	46.1	38.6	30.4	46.7	36.1	30.5	41.7
**CEAP 4**												
Dynamometry	8.4	*	*	10.4	*	*	7.3	*	*	8.1	*	*
Dorsiflexion	10	*	*	15	*	*	15	*	*	20	*	*
Plantar flexion	45	*	*	45	*	*	45	*	*	50	*	*
Leg circumference	29.3	*	*	29.2	*	*	29.2	*	*	28.5	*	*
Adipometry	25	*	*	20	*	*	20	*	*	18	*	*
**CEAP 5**												
Dynamometry	8.4	*	*	7.6	*	*	10.0	*	*	8.5	*	*
Dorsiflexion	10	*	*	10	*	*	10	*	*	13	*	*
Plantar flexion	35	*	*	40	*	*	45	*	*	50	*	*
Leg circumference	38.7	*	*	38.7	*	*	38.8	*	*	38.8	*	*
Adipometry	50	*	*	50	*	*	40	*	*	50	*	*

CEAP = Clinical, Etiology, Anatomy, and Pathophysiology; M = mean; CI = confidence interval; L = lower limit; U = upper limit.

* Number of patients insufficient to calculate confidence interval.

## DISCUSSION

The results show that the profile and characteristics of the patients are in line with what is described in the literature, as are their venous disease grades. According to a 2017 study by Santler and Goerge,^3^ the CEAP stages most often found in the population are C2 and C3.^11^^,^^12^

A 12-week treatment protocol (24 sessions) was applied, but the majority of eligible patients did not adopt a positive attitude to performing the exercises. Identification of their reasons for not participating could contribute to making the program more accessible, offering additional different locations and a wider selection of times and days to attend the group sessions.

The attendance rate at exercise sessions was 78.8%, which is very similar to the attendance rate observed in a 2018 study by Klonizakis et al.,^13^ who administered a 12-week treatment protocol (36 sessions) to C6 patients. Due to the Covid-19 pandemic, we had a considerable rate of loss to follow-up (attendance at fewer than 60% of the total number of sessions, thus these patients had to be excluded from the analysis) and enrollment of new groups was suspended.

It can be observed that when patients were subdivided by CEAP stage, their results varied according to CVI grades, which is because muscle pump dysfunction increases depending on the degree of compromise due to the disease, as explained by Back et al.^14^ The increase in muscle strength observed in this study confirms findings by Ercan et al.^15^ and Padberg et al.^16^ who administered physical exercise protocols for 12 weeks and 6 months respectively. It can therefore be concluded that physical exercise has a positive impact on muscle strength, which is generally compromised in people with CVI, helping to attenuate progression of the disease, since it reduces the process of muscle weakening that is linked to the process of chronic venous ulceration^17^ and improves venous pump function. For these effects to be lasting, exercise programs must have a minimum duration of 3 weeks and can be continued for up to 6 months.^7^^,^^18^

In addition to lack of strength, reduced ankle movement amplitude also impacts on venous pump function. In this study, physical exercise proved effective for increasing amplitude of both dorsiflexion and plantar flexion, as observed by Ercan in 2018.^15^ However, in 2018, Klonizakis et al.^13^ observed increased amplitude at a 12-week assessment, but amplitude had reduced at a 1-year assessment.

CVI is a pathology that reduces venous return, causing edema that results in increased limb circumference.^18^ The physical exercise intervention resulted in a reduction in leg circumference measurements, as was observed by Quilici et al.,^19^ and Kravtsov.^20^ In contrast, in 2006, Meyer^21^ found that the combination of lymphatic drainage and compression therapy resulted in a greater reduction in the circumference of the leg, whereas exercise alone did not provoke much change.

We observed reductions in anthropometric calf measurements, demonstrating that physical exercise reduces patients’ adipose tissue, similar to what was observed by Klonizakis et al.,^13^ who measured the reduction in terms of a muscle mass index, pointing out that reducing obesity can make a positive contribution to CVI treatment.^22^^,^^23^

Our study has a limitation caused by the low rate of adherence to the physical exercise intervention (13/69-18.8% - Figure 1), since treatment takes a long time and patients need to travel to attend. The same difficulty has been reported by other researchers, such as Klonizakis et al.^13^ The low rate of adherence to the physical exercise intervention meant that the minimum sample size of 42 limbs was not achieved.

Administration of exercise protocols should be considered as a treatment method, since its positive impact on risk factors and on functions that are impaired by the pathology can contribute to improving prognosis and reducing progression of CVI.
