# Supplementary material for: Effects of strengthening the surae triceps muscle on venous pump function in chronic venous insufficiency
Source: J Vasc Bras. 2021 Aug 16;20:e20200197. doi: 10.1590/1677-5449.200197 (PMC8372291; doi:10.1590/1677-5449.200197)
Supplement: Translation

# Translation: Efeitos do fortalecimento muscular do tríceps sural na função da bomba venosa na insuficiência venosa crônica

**Authors:** Ana Carla Schmidt, Luisa Pereira de Oliveira Zanetti Gomes, Camila Martins Marinelli, Ricardo Zanetti Gomes

**Affiliations:** 1 Centro de Ensino Superior dos Campos Gerais, Ponta Grossa PR, Brasil.; 2 Universidade Estadual de Ponta Grossa – UEPG, Ponta Grossa PR, Brasil.; 3 Universidade da Região de Joinville – UNIVILLE, Joinville, SC, Brasil.

**Keywords:** insuficiência venosa crônica, exercício físico, atividade física

## Abstract

**Contexto:**

A insuficiência venosa crônica (IVC) é uma doença comum, uma disfunção da bomba muscular da panturrilha que repercute na hemodinâmica das estruturas.

**Objetivos:**

Analisar os efeitos dos exercícios de fortalecimento muscular da panturrilha na hemodinâmica venosa em pacientes com IVC.

**Métodos:**

O estudo contou com a participação de 13 pacientes e 25 membros inferiores com IVC, classificados entre C1 e C5 da classificação Clínica, Etiologia, Anatomia e Fisiopatologia (CEAP), oriundos do ambulatório de cirurgia vascular do Hospital Universitário Regional dos Campos Gerais. As variáveis analisadas foram dinamometria isométrica, goniometria, perimetria, adipometria no início, primeiro mês, segundo mês e final da aplicação do protocolo de exercícios.

**Resultados:**

Os valores da dorsiflexão e da plantiflexão aumentaram 5º (p < 0,001). Na adipometria notou-se uma redução de 5 mm (p < 0,001). Quando separados em grupos pela CEAP, na C2 houve aumento de 5º na dorsiflexão (p = 0,02) e na plantiflexão (p < 0,001); na C3 houve aumento de 5º na dorsiflexão (p = 0,003) e redução de 1 mm na adipometria (p < 0,004); na C1 houve aumento da perimetria de 1,2 cm (p = 0,04).

**Conclusões:**

A aplicação de protocolos de exercícios deveria ser considerada como uma forma de tratamento para IVC, pois impacta positivamente em fatores de risco e nas funções que estão deterioradas pela patologia.

## INTRODUÇÃO

A insuficiência venosa crônica (IVC) é uma doença evolutiva definida por alterações do fluxo sanguíneo das veias, sendo que para seu desenvolvimento é necessária a combinação entre refluxo venoso, obstrução venosa e bomba muscular da panturrilha ineficiente. As veias da panturrilha e os tecidos circundantes formam a bomba muscular que atua na drenagem do sangue venoso, por meio do movimento da articulação do tornozelo, e sua disfunção pode agravar a IVC devido à hipertensão venosa^1^^,^ 2.

Atualmente, existe uma ampla gama de tratamentos para a IVC, que tem como objetivo prevenir as sequelas e melhorar os sintomas e suas complicações. O tratamento pode ser feito por meio de procedimentos cirúrgicos, terapia de compressão e terapia farmacológica. Para incrementar essas opções, o exercício físico vem sendo estudado como forma de prevenção e reabilitação da IVC, desonerando a saúde pública com a redução dos gastos e aumento da produtividade desses pacientes^3^^-^ 5.

Os efeitos dos exercícios estruturados para os pacientes com disfunção venosa são a melhora da função muscular, com aumento da força e trofismo e da hemodinâmica venosa. Os programas de exercícios geralmente consistem em técnicas de alongamento e fortalecimento dos músculos dos membros inferiores, juntamente com exercícios aeróbicos^6^^-^ 8. Assim, o objetivo deste estudo foi analisar os efeitos dos exercícios de fortalecimento muscular da panturrilha na hemodinâmica venosa, comparando essa melhora entre tempo de intervenção e entre os diferentes grupos da classificação Clínica, Etiologia, Anatomia e Fisiopatologia (CEAP).

## MÉTODOS

Trata-se de um estudo experimental caracterizado como ensaio clínico não randomizado, sem grupo controle, em uma população com IVC. Foi realizado no ambulatório de cirurgia vascular do Hospital Universitário Regional dos Campos Gerais, onde os pacientes com IVC passam por atendimento médico, tendo sido aprovado pelo comitê de ética da instituição (número do parecer: 3653067). Foram incluídos pacientes com IVC classificados de C1 a C5 pela CEAP, de ambos os sexos, não submetidos à cirurgia convencional de varizes ou escleroterapia com espuma e que tinham 60% de presença nos grupos de fortalecimento da panturrilha. Foram excluídos indivíduos com disfunções ortopédicas, reumatológicas e/ou neurológicas de membros inferiores, úlceras venosas ou outras etiologias, com histórico recente de fraturas em membros inferiores e que não aceitaram participar da pesquisa.

### Recrutamento da amostra

Foi realizado um cálculo de tamanho de amostra, e, considerando um efeito esperado de 20%, poder de 80% e erro de 10%, calculou-se uma amostra mínima de 42 membros. Realizou-se então uma busca nos prontuários do ambulatório vascular, sendo que os pré-selecionados foram contatados via telefone (Figura 1). O formulário elaborado pelos próprios autores tinha como finalidade identificar as principais características da população estudada, bem como informações sobre a doença. Ele foi inicialmente aplicado junto aos 69 participantes e os exames físicos foram reaplicados em 13 pacientes que completaram todas as etapas do estudo, perfazendo 25 membros inferiores (um membro foi excluído por cirurgia). Nessa fase, realizou-se um cálculo de poder amostral, sendo que para a mesma expectativa de sucesso de 20%, com amostral de 25 membros, o poder da amostra foi de 64%.

**Figura 1:** Recrutamento da amostra.

### Exames físicos

Os pacientes selecionados passaram pelos exames de força muscular do tríceps sural realizado pelo dinamômetro isométrico portátil do Manual Muscle Teste: Model 01165®. Para essa medição, o paciente foi posicionado em decúbito dorsal, com quadril e joelhos estendidos e tornozelos na posição neutra, e o dinamômetro sendo posicionado na região proximal das articulações metatarso falangianas na planta do pé. Nessas condições, o paciente deveria realizar a plantiflexão contra a resistência do examinador. As medidas foram repetidas três vezes com intervalos de 15 segundos e calculada a média entra elas, sendo o resultado obtido em quilograma-força.

A amplitude de movimento da articulação do tornozelo foi medida pela goniometria manual, cujo valor resultou da soma da máxima e voluntária da flexão plantar mais a dorsal a partir da medida neutra de 90º. Os valores esperados eram de 45º para a flexão plantar e de 20º para a dorsal, resultando num total de 65º. As medições, tanto da dorsiflexão quanto da flexão plantar, foram realizadas com o paciente em decúbito dorsal, os joelhos fletidos em 30º e o pé em posição anatômica.

A circunferência da perna foi medida através da perimetria com fita métrica e o paciente em posição ortostática, com marcação de pontos separados por 10 cm desde a borda inferior da patela até a região maleolar em ambos os membros inferiores. As medidas foram realizadas no período da tarde e a unidade utilizada foi centímetros.

Na medição do percentual de gordura da panturrilha foi utilizada a adipometria. Nesse caso, o avaliador deve pinçar a dobra cutânea com os dedos cerca de 1,5 cm do ponto de aplicação do adipômetro, sendo o ponto de aplicação do equipamento a máxima circunferência da perna com o paciente sentado. Essa avaliação foi repetida três vezes para se obter uma média final em milímetros.

Os dados foram obtidos no início da intervenção (1º atendimento), após um mês de intervenção (9º atendimento), após dois meses de intervenção (17º atendimento) e ao final da intervenção (25º atendimento), individualmente.

### Protocolo de exercícios

A intervenção perdurou 3 meses, totalizando 24 atendimentos: dois atendimentos semanais, por 30 minutos, cada exercício realizado em três a cinco séries de 10 a 12 repetições, com 60 segundos de intervalo entre as séries, conforme os parâmetros de treinamento descritos por Schoenfeld^9^.

Os exercícios foram realizados em cadeia cinética aberta e fechada, por meio da flexão plantar e dorsal da articulação do tornozelo. Cada movimento prezou a cadência das fases concêntrica, excêntrica e isométrica, sendo esses intensificadores do exercício.

### Análise estatística

Inicialmente, procedeu-se a análise descritiva das variáveis relacionadas aos pacientes com estimativas de frequências simples. As variáveis dinamometria, dorsiflexão, plantiflexão, perimetria e adipometria foram analisadas com estimativa de média, mediana, desvio padrão, percentil 25% e 75% em cada momento de acompanhamento.

Em seguida, verificou-se a aderência das mesmas quanto à distribuição normal pelo teste Shapiro-Wilk e, como não apresentaram distribuição normal, utilizou-se a abordagem não paramétrica dos dados. A diferença entre os momentos de acompanhamento foi testada com o teste de Friedman seguido de *post-hoc* de comparações múltiplas com correção de Bonferroni. Os testes foram considerados significativos quando p < 0,05 e as análises foram realizadas no SPSS 21.0 (IBM, 2012)^10^.

## RESULTADOS

Neste estudo, 13 participantes (25 membros inferiores) completaram 24 sessões de intervenção fisioterapêutica e realizaram quatro avaliações. A amostra foi composta por mulheres com idade entre 38 a 70 anos e diagnosticadas com IVC em média há 6,4 +- 7,5 anos. Os dados usados para identificar os indivíduos, patologia associadas, medicamentos utilizados e estilo de vida estão dispostos na Tabela 1.

**Tabela 1:** Identificação da amostra.

**Características**			**n**	**%**
Sexo	Feminino		13	100,0%
Faixa etária	Até 50 anos		6	46,2%
	Acima de 50 anos		7	53,8%
Tempo de diagnóstico	Até 5 anos		9	69,2%
	Mais de 5 anos		4	30,8%
Meia de compressão	Sim		6	46,2%
	Não		7	53,8%
Patologia associada	Diabetes *mellitus*		5	38,5%
	Hipertensão arterial		4	30,8%
	Distúrbios de tireoide		3	23,1%
	Dislipidemia		2	15,4%
	Outros		7	53,8%
Fármacos usados	Diosmina + hesperidina		5	38,5%
	Levotiroxina		5	38,5%
	Metformina		3	23,1%
	Losartana		2	15,4%
	Sinvastatina		2	15,4%
	Ácido acetilsalicílico		2	15,4%
	Omeprazol		2	15,4%
	Outros		15	115,5%
Estilo de vida	Tabagista	Sim	1	7,7%
		Não	8	61,5%
		Ex-tabagista	4	30,8%
	Etilista	Não	12	92,3%
		Ex-etilista	1	7,7%
	Atividade física	Sedentário	7	53,8%
		Ativo	6	46,2%
	Frequência da atividade física	1 dia	1	7,7%
		3 dias	3	23,1%
		7 dias	2	15,4%

A avaliação da doença venosa de cada paciente foi realizada conforme a classificação CEAP. O agrupamento das participantes ocorreu pelo aspecto clínico (C), sendo observado na Tabela 2 a disposição dos indivíduos em cada grau. Utilizando essa divisão dos grupos conforme a classificação da CEAP, verificou-se que não houve diferença estatística significativa intra e entre os grupos da CEAP quanto ao ganho de força muscular do tríceps sural, conforme demonstrado na Tabela 3. Porém, ao analisar o resultado geral da dinamometria (Tabela 4) percebeu-se que, mesmo não sendo significativo, houve um aumento da força entre a avaliação inicial e final.

**Tabela 2:** Classificação da IVC pela CEAP.

CEAP	n	%
**C1**	5	20,0%
**C2**	11	44,0%
**C3**	7	28,0%
**C4**	1	4,0%
**C5**	1	4,0%

CEAP = Clínica, Etiologia, Anatomia e Fisiopatologia.

**Tabela 3:** Valores da mediana e intervalo interquartil da dinamometria em pacientes submetidos ao exercício acompanhados por 3 meses e valores de p do teste de diferença entre os mesmos.

**CEAP**	**n**	**Dinamometria - kgf**												**p-valor entre tempos** ^*^
		**Inicial**			**1º mês**			**2º mês**			**Final**			
		**MD**	**Perc 25%**	**Perc 75%**	**MD**	**Perc 25%**	**Perc 75%**	**MD**	**Perc 25%**	**Perc 75%**	**MD**	**Perc 25%**	**Perc 75%**	
C1	5	7,3	6,9	8,0	7,5	7,5	8,2	7,8	7,7	7,8	7,5	7,3	7,8	0,246
C2	11	7,3	6,9	9,8	8,2	7,7	8,9	8,0	7,6	8,8	8,7	7,6	8,8	0,383
C3	7	9,3	6,8	10,1	8,2	6,3	9,2	8,7	7,0	10,0	9,0	7,5	9,6	0,136
C4	1	8,4	8,4	8,4	10,4	10,4	10,4	7,3	7,3	7,3	8,1	8,1	8,1	0,392
C5	1	8,4	8,4	8,4	7,6	7,6	7,6	10,0	10,0	10,0	8,5	8,5	8,5	0,392

MD = mediana; Perc = percentil.

* Teste de Friedman (análogo à ANOVA para medidas repetidas não paramétrico). Letras diferentes correspondem a diferenças estatisticamente significativas nas comparações dois a dois.

**Tabela 4:** Valores da mediana e intervalo interquartil geral em pacientes submetidos ao exercício acompanhados por 3 meses e valores de p do teste de diferença entre os mesmos.

**Geral**	**n**	**Inicial**			**1º mês**			**2º mês**			**Final**			**p-valor entre momentos** ^*^
		**MD**	**Perc 25%**	**Perc 75%**	**MD**	**Perc 25%**	**Perc 75%**	**MD**	**Perc 25%**	**Perc 75%**	**MD**	**Perc 25%**	**Perc 75%**	
Dinamometria (kgf)	25	8,0a	6,9	9,8	8,2a	7,5	8,9	8,0a	7,6	8,9	8,5a	7,5	8,8	0,266
Dorsiflexão (º)	25	15,0a	10,0	20,0	20,0ab	20,0	20,0	20,0b	20,0	20,0	20,0b	20,0	25,0	< 0,001
Plantiflexão (º)	25	40,0a	35,0	40,0	40,0a	40,0	40,0	40,0a	40,0	45,0	45,0b	42,0	50,0	< 0,001
Perimetria (cm)	25	30,2ª	29,3	33,0	31,8a	29,3	33,3	31,8a	29,0	33,3	30,8a	29,2	33,2	0,215
Adipometria (mm)	25	35,0a	30,0	45,0	32,0ab	30,0	40,0	30,0ab	30,0	40,0	30,0b	30,0	40,0	< 0,001

MD = mediana; Perc = percentil;

* Teste de Friedman (análogo à ANOVA para medidas repetidas não paramétrico). Letras diferentes correspondem a diferenças estatisticamente significativas nas comparações dois a dois.

A mesma análise foi realizada com a goniometria dividida em dorsiflexão e plantiflexão. Nos valores da dorsiflexão observou-se que os grupos C2 (n = 11) e o C3 (n = 7) tiveram mais benefícios com os exercícios propostos em relação aos demais grupos (Tabela 5). Esses resultados também foram observados na flexão plantar, como mostra a Tabela 6. Quando analisadas essas variáveis sem divisão dos grupos (Tabela 5), nota-se que a amplitude de movimento aumentou significativamente da primeira à última sessão (p < 0,001, ambos).

**Tabela 5:** Valores da mediana e intervalo interquartil da goniometria-dorsiflexão em pacientes submetidos ao exercício acompanhados por 3 meses e valores de p do teste de diferença entre os mesmos.

**CEAP**	**n**	**Dorsiflexão (º)**												**p-valor entre tempos** ^*^
		**Inicial**			**1º mês**			**2º mês**			**Final**			
		**MD**	**Perc 25%**	**Perc 75%**	**MD**	**Perc 25%**	**Perc 75%**	**MD**	**Perc 25%**	**Perc 75%**	**MD**	**Perc 25%**	**Perc 75%**	
C1	5	20,0	20,0	20,0	25,0	20,0	25,0	25,0	20,0	25,0	25,0	20,0	30,0	0,072
C2	11	15,0	10,0	20,0	20,0	20,0	20,0	20,0	20,0	20,0	20,0	20,0	25,0	0,002
C3	7	15,0	10,0	20,0	20,0	20,0	20,0	20,0	20,0	20,0	20,0	20,0	25,0	0,002
C4	1	10,0	10,0	10,0	15,0	15,0	15,0	15,0	15,0	15,0	20,0	20,0	20,0	0,392
C5	1	10,0	10,0	10,0	10,0	10,0	10,0	10,0	10,0	10,0	13,0	13,0	13,0	0,392

MD = mediana; Perc = percentil.

* Teste de Friedman (análogo à ANOVA para medidas repetidas não paramétrico). Letras diferentes correspondem a diferenças estatisticamente significativas nas comparações dois a dois.

**Tabela 6:** Valores da mediana e intervalo interquartil da goniometria-plantiflexão em pacientes submetidos ao exercício acompanhados por 3 meses e valores de p do teste de diferença entre os mesmos.

**CEAP**	**n**	**Plantiflexão (º)**												**p-valor entre tempos** ^*^
		**Inicial**			**1º mês**			**2º mês**			**Final**			
		**MD**	**Perc 25%**	**Perc 75%**	**MD**	**Perc 25%**	**Perc 75%**	**MD**	**Perc 25%**	**Perc 75%**	**MD**	**Perc 25%**	**Perc 75%**	
C1	5	40,0	30,0	40,0	40,0	35,0	40,0	40,0	35,0	40,0	40,0	40,0	40,0	0,067
C2	11	40,0	35,0	40,0	40,0	40,0	45,0	40,0	40,0	45,0	45,0	45,0	50,0	< 0,001
C3	7	40,0	30,0	40,0	40,0	40,0	40,0	40,0	40,0	40,0	45,0	45,0	50,0	0,003
C4	1	45,0	45,0	45,0	45,0	45,0	45,0	45,0	45,0	45,0	50,0	50,0	50,0	0,392
C5	1	35,0	35,0	35,0	40,0	40,0	40,0	45,0	45,0	45,0	50,0	50,0	50,0	0,392

MD = mediana; Perc = percentil.

* Teste de Friedman (análogo à ANOVA para medidas repetidas não paramétrico). Letras diferentes correspondem a diferenças estatisticamente significativas nas comparações dois a dois.

Em contrapartida aos demais resultados já apresentados, observou-se (Tabela 7) que os valores da perimetria aumentaram significativamente para o grupo C1 (n = 5) ao final das 24ª sessões em comparação com os outros grupos. Em uma visão geral dos resultados da perimetria (Tabela 4) houve um aumento nos valores, mas não estatisticamente significativo.

**Tabela 7:** Valores da mediana e intervalo interquartil da perimetria em pacientes submetidos ao exercício acompanhados por 3 meses e valores de p do teste de diferença entre os mesmos.

	**n**	**Perimetria (cm)**												**p-valor entre tempos** ^*^
		**Inicial**			**1º mês**			**2º mês**			**Final**			
		**MD**	**Perc 25%**	**Perc 75%**	**MD**	**Perc 25%**	**Perc 75%**	**MD**	**Perc 25%**	**Perc 75%**	**MD**	**Perc 25%**	**Perc 75%**	
C1	5	29,5	29,1	32,2	31,8	28,8	32,7	31,8	29,3	32,7	30,7	28,8	31,7	0,045
C2	11	29,8	29,0	31,5	30,3	29,3	32,7	29,2	28,5	33,0	29,3	29,2	32,2	0,197
C3	7	32,7	30,2	33,7	33,3	30,0	33,8	33,3	29,8	33,5	32,0	30,3	33,5	0,427
C4	1	29,3	29,3	29,3	29,2	29,2	29,2	29,2	29,2	29,2	28,5	28,5	28,5	0,392
C5	1	38,7	38,7	38,7	38,7	38,7	38,7	38,8	38,8	38,8	38,8	38,8	38,8	0,392

MD = mediana; Perc = percentil.

* Teste de Friedman (análogo à ANOVA para medidas repetidas não paramétrico). Letras diferentes correspondem a diferenças estatisticamente significativas nas comparações dois a dois.

Na adipometria, o grupo C3 apresentou uma redução significativa nos valores obtidos (p = 0,004). O mesmo ocorreu no grupo C4, embora não tenha sido significativa (Tabela 8). Observou-se que o percentual de gordura da panturrilha nos 25 membros diminuiu após a intervenção (Tabela 4) (p < 0,001).

**Tabela 8:** Valores da mediana e intervalo interquartil da adipometria em pacientes submetidos ao exercício acompanhados por 3 meses e valores de p do teste de diferença entre os mesmos.

**CEAP**	**n**	**Adipometria (mm)**												**p-valor entre tempos** ^*^
		**Inicial**			**1º mês**			**2º mês**			**Final**			
		**MD**	**Perc 25%**	**Perc 75%**	**MD**	**Perc 25%**	**Perc 75%**	**MD**	**Perc 25%**	**Perc 75%**	**MD**	**Perc 25%**	**Perc 75%**	
C1	5	35,0a	30,0	40,0	32,0a	30,0	40,0	32,0a	30,0	40,0	40,0a	30,0	40,0	0,194
C2	11	30,0a	26,0	35,0	30,0a	25,0	35,0	30,0a	25,0	32,0	30,0a	25,0	32,0	0,153
C3	7	36,0a	35,0	51,0	36,0ab	35,0	45,0	35,0ab	30,0	48,0	35,0b	30,0	40,0	0,004
C4	1	25,0a	25,0	25,0	20,0a	20,0	20,0	20,0a	20,0	20,0	18,0a	18,0	18,0	0,392
C5	1	50,0a	50,0	50,0	50,0a	50,0	50,0	40,0a	40,0	40,0	50,0a	50,0	50,0	0,392

MD = mediana; Perc = percentil.

* Teste de Friedman (análogo à ANOVA para medidas repetidas não paramétrico). Letras diferentes correspondem a diferenças estatisticamente significativas nas comparações dois a dois.

A análise dos resultados estatisticamente significativos permitiu visualizar uma diferença entre os momentos de acompanhamento nas variáveis dorsiflexão, plantiflexão e adipometria. A dorsiflexão aumentou significativamente no 2º mês e no final (p = 0,016 e p < 0,001, respectivamente). A plantiflexão foi significativamente maior no momento final do que nos momentos inicial, 1º mês e 2º mês (p < 0,001, p = 0,003 e p = 0,037, respectivamente). A adipometria foi significativamente menor no momento final do que no momento inicial (p = 0,03). Os intervalos de confiança dos achados estão na Tabela 9.

**Tabela 9:** Tabela com os intervalos de confianças das variáveis analisadas.

	**inicial**			**1° mês**			**2° mês**			**Final**		
	**M**	**IC95%**		**M**	**IC95%**		**M**	**IC95%**		**M**	**IC95%**	
		**inf**	**sup**		**inf**	**sup**		**inf**	**sup**		**inf**	**sup**
**GERAL**												
Dinamometria	8,4	7,7	9	8,3	7,7	8,8	8,4	7,9	9,0	8,4	8	8,8
Dorsiflexão	15,8	13,4	18,2	19,6	17,8	21,4	20,4	18,8	22	22,4	20	24,8
Plantiflexão	37,6	35,7	39,5	40,4	38,6	42,2	41,4	39,8	43	45,3	43,5	47,1
Perimetria	31,6	30,3	32,9	31,9	30,7	33,1	31,6	30,3	32,9	31,5	30,2	32,7
Antropimetria	35,8	31,6	39,9	34,1	30,8	37,5	33,4	30,1	36,6	33,7	30,2	37,3
**CEAP 1**												
Dinamometria	8,0	5,9	10,0	8,4	6,1	10,6	8,5	6,1	10,9	8,0	6,4	9,6
Dorsiflexão	21,0	14,2	27,8	24,0	18,8	29,2	24,0	18,8	29,2	27,0	16,6	37,4
Plantiflexão	36,0	29,2	42,8	37,0	31,4	42,6	38,0	30,9	45,1	40,0	35,6	44,4
Perimetria	30,7	27,2	34,3	31,3	27,7	34,9	31,4	27,9	34,9	30,6	27,4	33,8
Antropimetria	36,0	27,9	44,1	34,4	28,0	40,8	34,4	28,0	40,8	36,6	29,0	44,2
**CEAP 2**												
Dinamometria	8,2	7,0	9,5	8,3	7,8	8,7	8,2	7,6	8,8	8,4	7,9	8,9
Dorsiflexão	15,5	11,6	19,3	19,1	16,6	21,6	20,5	18,6	22,3	21,8	18,4	25,3
Plantiflexão	38,2	35,9	40,4	41,8	38,4	45,3	42,7	40,4	45,0	45,9	43,4	48,4
Perimetria	31,1	28,9	33,3	31,4	29,4	33,4	30,9	28,8	33,0	31,1	29,0	33,2
Antropimetria	31,1	25,8	36,4	30,5	26,4	34,6	30,2	26,2	34,2	30,8	25,1	36,6
**CEAP 3**												
Dinamometria	8,8	7,4	10,3	7,9	6,5	9,3	8,7	7,2	10,2	8,7	7,7	9,7
Dorsiflexão	14,3	10,1	18,4	19,2	17,5	21,0	20,0	*	*	21,7	19,5	23,9
Plantiflexão	37,1	31,9	42,4	40,0	*	*	40,7	39,0	42,5	46,7	43,7	49,7
Perimetria	32,3	29,9	34,5	32,6	30,4	34,7	32,2	29,9	34,4	32,0	29,8	34,2
Antropimetria	42,4	32,1	52,7	39,4	32,7	46,1	38,6	30,4	46,7	36,1	30,5	41,7
**CEAP 4**												
Dinamometria	8,4	*	^*^	10,4	*	*	7,3	*	*	8,1	*	*
Dorsiflexão	10	*	*	15	*	*	15	*	*	20	*	*
Plantiflexão	45	*	*	45	*	*	45	*	*	50	*	*
Perimetria	29,3	*	*	29,2	*	*	29,2	*	*	28,5	*	*
Antropimetria	25	*	*	20	*	*	20	*	*	18	*	*
**CEAP 5**												
Dinamometria	8,4	*	*	7,6	*	*	10,0	*	*	8,5	*	*
Dorsiflexão	10	*	*	10	*	*	10	*	*	13	*	*
Plantiflexão	35	*	*	40	*	*	45	*	*	50	*	*
Perimetria	38,7	*	*	38,7	*	*	38,8	*	*	38,8	*	*
Antropimetria	50	*	*	50	*	*	40	*	*	50	*	*

CEAP = Clínica, Etiologia, Anatomia e Fisiopatologia; M = média; IC = intervalo de confiança.

* Número insuficiente de pacientes para cálculo.

## DISCUSSÃO

Com os resultados obtidos pode-se observar que o perfil e características dos pacientes estão de acordo com o que se encontra na literatura, bem como os graus da doença venosa. Segundo Santler & Goerge, em 2017^3^, os estágios mais encontrados na população são C2 e C3 da classificação CEAP^11^^,^ 12.

Foi aplicado um protocolo de tratamento de 12 semanas (24 atendimentos), e a maioria dos pacientes elegíveis não teve uma atitude positiva em relação à realização dos exercícios. A identificação das razões para a não participação poderá ajudar o programa a ser mais acessível, com a oferta de mais locais para a prática e disponibilização de mais horários e dias para os grupos.

A frequência aos exercícios foi de 78,8%, ficando bem próxima à frequência obtida no estudo de Klonizakis et al.^13^, com seu protocolo de tratamento de 12 semanas (36 atendimentos) em pacientes C6. Devido à pandemia da COVID-19, tivemos uma perda importante de seguimento (frequência inferior a 60% do número total de atendimentos), bem como a interrupção de novos grupos.

Pode-se observar que conforme a divisão da CEAP os resultados variam de acordo com a classificação apresentada, pois dependendo do grau de comprometimento da doença há maior disfunção da bomba muscular, como explicam Back et al.^14^. O aumento da força muscular identificado neste estudo corrobora os achados de Ercan et al.^15^, e Padberg et al.^16^, com a aplicação do protocolo de exercício físico durante as 12 semanas e 6 meses respectivamente. Com isso, pode-se afirmar que o exercício físico tem impacto sobre a força muscular, geralmente comprometida em pessoas com IVC, auxiliando a diminuir a progressão da doença, uma vez que reduz o processo de fragilidade muscular relacionado ao processo de ulcerações venosas crônicas^17^ e melhora a função da bomba venosa. Para que esses efeitos sejam duradouros, os programas de exercícios devem ter no mínimo 3 semanas e podem ser mantidos por até 6 meses^7^^,^ 18.

Além da falta de força, a amplitude do movimento do tornozelo também interfere na função da bomba venosa. Neste estudo, o aumento da amplitude com o exercício físico demostrou ser eficaz, assim como observado por Ercan^15^, tanto na dorsiflexão quanto na flexão plantar. Klonizakis et al.^13^, no entanto, observou em avaliação depois de 12 semanas de intervenção um aumento da amplitude, mas, depois de 1 ano, uma redução dessa amplitude.

A IVC é uma patologia que reduz o retorno venoso, causando edema que leva ao aumento da circunferência dos membros^18^. A aplicação de exercícios físicos resultou em uma redução dos valores da perimetria, assim como encontrado em Quilici^19^, e Kravtsov^20^. Por outro lado, Meyer^21^, identificou que a associação da drenagem linfática e a pressoterapia resultaram em uma redução maior da circunferência da perna, enquanto só o exercício não provocou grande alteração.

Observamos a redução das medidas antropométricas das panturrilhas, demonstrando que o exercício físico reduz o tecido adiposo nos pacientes, de forma semelhante ao que encontrou Klonizakis et al.^13^, que mediu a redução por meio do índice de massa muscular, lembrando que a redução da obesidade pode contribuir positivamente no tratamento da IVC^22^^,^ 23.

Nosso estudo apresenta uma limitação referente à taxa de adesão ao exercício físico (13/69-18,8% - Figura 1), uma vez que o tratamento é de longa duração e demanda tempo para o deslocamento e para a sua realização. Essa mesma dificuldade foi encontrada por outros pesquisadores, como Klonizakis et al.^13^. A baixa taxa de adesão ao exercício físico fez com que o número mínimo calculado da amostra de 42 membros não fosse atingido.

A aplicação de protocolos de exercícios deve ser considerada como uma forma de tratamento, pois seu impacto positivo sobre fatores de risco e nas funções que estão deterioradas pela patologia pode contribuir para uma melhora no prognóstico e redução na evolução da IVC.
